# Engineering Optimized EV-Mimetic Carriers for Efficient Tumor-Targeted Delivery of Functional RNA Nanoparticles

**DOI:** 10.1021/acsnanomed.6c00004

**Published:** 2026-02-20

**Authors:** Nathalia Leal Dovaizem, Laura P. Rebolledo, Anh Ha, Roger Chammas, Luciana Nogueira de Sousa Andrade, Kirill A. Afonin

**Affiliations:** † Center for Translational Research in Oncology (LIM/24), Instituto do Cancer do Estado de Sao Paulo, Hospital das Clinicas HCFMUSP, Faculdade de Medicina, Universidade de São Paulo, São Paulo 01246-000, Brazil; ‡ Comprehensive Center for Precision Oncology (C2PO), Universidade de São Paulo, São Paulo 01246-000, Brazil; § Department of Chemistry, 14727 University of North Carolina at Charlotte, Charlotte, North Carolina 28223, United States

**Keywords:** Extracellular Vesicles, EV Mimetics, Nucleic Acid Nanoparticles, RNAi, New Approach Methodologies

## Abstract

The therapeutic potential of RNAi is limited by the lack of safe, scalable, and efficient delivery systems. Although extracellular vesicles (EVs) are promising carriers for RNA therapeutics, poor scalability and low cargo-loading efficiency constrain their clinical translation. Here, we introduce fully synthetic EV-mimetic (EVM) carriers for the delivery of RNAi-inducing RNA nanoparticles to tumor cells. We systematically evaluated four different EVM cargo-loading strategies using representative RNA nanoparticles and appropriate controls. For each strategy, EVM size, concentration, and cargo encapsulation efficiency were assessed. Cellular uptake and RNA delivery were examined in melanoma 3D spheroids and compared with natural tumor-derived EVs, with EVMs demonstrating superior delivery efficiency. Functional RNAi activation was evaluated in tumor monolayers and 3D spheroids, where EVMs exhibited effective gene silencing with lower toxicity than conventional lipid-based carriers. Overall, our findings establish EVMs as safe and scalable biomimetic platforms for therapeutic nucleic acid delivery and identify optimal loading strategies and RNA nanoparticle designs for achieving maximal gene-silencing.

## Graphical abstract

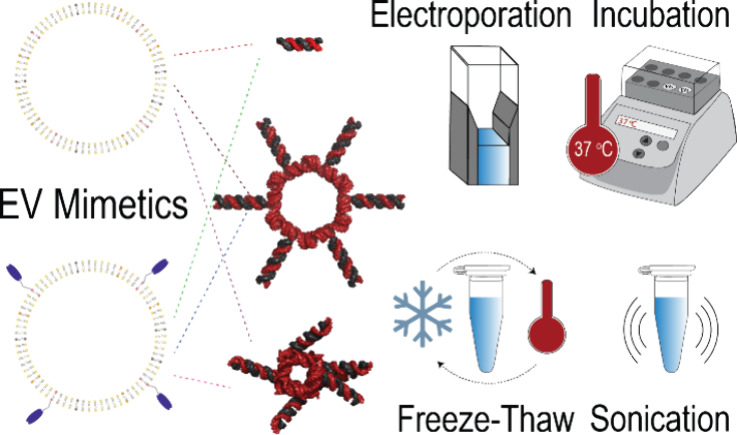

## Introduction

RNA interference (RNAi) has emerged as a powerful therapeutic strategy for post-transcriptional gene silencing through the functional attenuation or degradation of target mRNAs.^1^ In recent years, several RNAi-based drugs have received regulatory approval for hereditary transthyretin,^2^ acute-intermittent porphyria,^3^ hyperoxaluria type 1,^4,5^ and hypercholesterolemia,^6^ and many others are in preclinical and clinical development for the treatment of diverse diseases, including cancer.^7–9^ Beyond these advances, the success of mRNA vaccines against COVID-19 boosted the acceptance of RNA-based therapies and highlighted their broad clinical potential.^10^ In oncology, RNAi has been investigated as first- or second-line therapy, being commonly used as an adjuvant to inhibit genes associated with tumor progression and drug resistance.^11–14^

A major challenge in overcoming tumor resistance is its multifactorial nature, as it is often driven by the dysregulated expression of multiple genes and pathways.^15,16^ In this scenario, the development of nucleic acid nanoparticles (NANPs), a next-generation RNAi platform, represents an advantage as they can be designed to carry multiple RNAi inducers in a single, well-defined architecture, enabling multitarget combinatorial gene silencing.^17,18^ NANPs can be designed in different shapes and depending on the nucleic acid composition and dimensionality, they can either avoid or stimulate innate immune signaling pathways, thus boosting antitumor immune responses and improving therapeutic outcomes.^19–21^ Despite these promising features, their clinical translation remains challenged by the need for effective delivery systems that can protect the cargo from nucleases while achieving selective tissue uptake and minimizing off-target effects. Viral vectors and lipid-based nanoparticles (LNPs) have been used as RNA therapeutic carriers, but their clinical applications are also limited by uncontrolled immunogenicity, safety concerns, restricted endosomal escape, and tissue tropism.^22–24^

As an alternative, extracellular vesicles (EVs) have emerged as highly attractive carriers, due to their superior biocompatibility, low immunogenicity, and natural tropism.^25^ They are nanosized lipid bilayer vesicles secreted by virtually all cell types, carrying diverse biomolecules (e.g., proteins, lipids, and nucleic acids) and playing key roles in cell-to-cell communication.^26^ Several studies have demonstrated that EVs outperform conventional liposomes and viral systems in terms of uptake and delivery efficiency, both *in vitro* and *in vivo*.^27–31^ Additionally, a recent phase I clinical trial using EVs loaded with siRNAs targeting KRAS^G12D^ in pancreatic cancer demonstrated the feasibility and safety of EV-based RNA delivery in humans, further supporting their therapeutic potential.^32^ However, their broader clinical translation remains limited by manufacturing challenges, including low yields from standard isolation methods, biological heterogeneity driving batch-to-batch variability, and generally poor cargo loading efficiency.^33^ To overcome some of these limitations, synthetic EV mimetics (EVMs) have been proposed as alternatives to recapitulate the functional properties of natural EVs while allowing controlled composition and scalable production.^34^ These synthetic systems are designed to mimic the lipid composition of natural EVs and can be functionalized with EV-associated tetraspanins, shown to play important roles in EV uptake and cargo release.^35,36^ Additionally, EVMs can be decorated with target peptides for delivery to specific cells and tissues.^37^

Moreover, a remaining challenge for clinical application of both natural EVs and synthetic EVMs is the efficient loading of therapeutic cargo.^38,39^ Among the methods currently explored, passive incubation, electroporation, sonication, and freeze–thaw cycling have been reported, each presenting a trade-off in loading efficiency, vesicle integrity, and cargo stability.^40^ While these approaches have mostly been evaluated individually for conventional siRNA encapsulation in natural EVs, studies addressing their performance side-by-side, with structured RNA nanoassemblies and EVMs are underexplored.

Here, we report on the use of synthetic EVMs optimized for efficient delivery of various RNAi inducers to tumor cells grown in 3D spheroids. The uptake and cargo delivery into tumor spheroids were compared between EVMs (with or without CD63 and CD9 peptide decoration) and natural EVs derived from SKMel-28, WM-1366, and A375 human melanoma cells. Spheroids treated with EVMs loaded with fluorescently labeled cargo exhibited a higher percentage of EVM-positive cells compared to those treated with natural EVs, reflecting more efficient cargo loading into synthetic EVMs. Moreover, compared to melanoma-derived EVs, ^CD63+/CD9+^EVMs did not impair innate immune cell proliferation and viability, highlighting their potential for clinical translation. Additionally, by comparing four different loading strategies (incubation, electroporation, sonication, and freeze–thaw) for their ability to encapsulate three distinct functional RNA architectures, we showed that electroporation was the most efficient method for achieving functional cargo transfer and GFP silencing in MDA-MB-231-eGFP triple-negative breast cancer cells across all nanostructures. For cells grown in monolayers, EVMs loaded via electroporation exhibited similar silencing efficacy and reduced toxicity compared to conventional lipid-based carriers (i.e., Lipofectamine 2000 and DOTAP/DOPE). In 3D spheroids, EVMs outperformed lipid carriers while maintaining similar toxicity profiles, highlighting the potential of EVM formulations as an alternative delivery methodology for therapeutic nucleic acids.

## Materials and Methods

### Assembly and Functionalization of EVMs

Synthetic EVMs were prepared through a multilamellar vesicle (MLV) to small unilamellar vesicle (SUV) transition followed by emulsification, as previously published^34^ with certain modifications. Briefly, lipids were dissolved in chloroform at the following molar ratios: 55% cholesterol, 29% 1,2-dioleoyl-*sn*-glycero-3-phosphocholine (DOPC), 10% 1,2-dioleoyl-*sn*-glycero-3-phospho-l-serine (DOPS), 4% sphingomyelin (SM), and 2% 1,2-dioleoyl-*sn*-glycero-3-[(N-(5-amino-1-carboxypentyl)­iminodiacetic acid)­succinyl] nickel salt (DGS-NTA­(Ni)). The organic solvent was evaporated to form a dry lipid film, which was subsequently rehydrated with 10 mM MgCl_2_ in phosphate-buffered saline (PBS) to a final lipid concentration of 6 mM. The suspension was shaken for 5 min to obtain MLVs. SUVs were generated from MLVs by probe sonication (5 s on/off cycles, 15% amplitude, 15 min) in an ice-cold bath. The resulting suspension was centrifuged at 100,000*g* for 30–70 min, and the pellet was resuspended in PBS/MgCl_2_. Next, SUVs were emulsified with a fluorosurfactant-containing FC-40 oil phase at a 2:1 oil-to-water ratio. A triblock surfactant was added to a final concentration of 1.25 mM, and the mixture was emulsified at maximum speed for 60 s, followed by incubation for at least 2 h at 4 °C. The excess oil phase was removed, PBS and perfluoro-1-octanol (PFO) were added to the droplet mixture at a 1:1:1 ratio, and incubated for 30 min at 4 °C. The EVM-containing aqueous phase was collected, centrifuged at 100,000*g* for 30–70 min, and the resulting pellet was resuspended in PBS containing 150 mM NaCl, pH 7.6. For surface functionalization, EVMs were incubated with His-tagged peptides at a protein-to-lipid molar ratio of 1:100 for 1 h at 37 °C. The vesicles were then washed with PBS containing 0.1% BSA and centrifuged at 100,000*g* for 30–70 min. The pellet was resuspended in PBS. EVMs were stored at 4 °C for up to 1 week or at −80 °C for long-term preservation.

### Isolation of Tumor-Derived EVs

EVs were isolated by differential ultracentrifugation,^41^ followed by size exclusion chromatography (SEC). Briefly, the conditioned media of melanoma cells grown in monolayers, at 80% confluency, were collected and centrifuged at 300*g* for 10 min, followed by 2000*g* for 10 min, both at room temperature. Next, the supernatants were ultracentrifuged at 10,000*g* for 30 min at 4 °C, followed by 100,000*g* for 2 h at 4 °C (Beckman Coulter Optima XE-90, rotor SW28). EV pellets were washed with PBS, recentrifuged at 100,000*g* for 2 h at 4 °C, resuspended in 500 μL of PBS, and loaded onto qEV 70 nm columns (Izon). Next, 8 mL of PBS was added, and the flowthrough was collected in 7 fractions of 1 mL each. EVs-enriched fractions (2 to 4) were pooled and stored at −80 °C for further experiments. EVs characterization was conducted according to the 2023 consensus guidelines on Minimal Information for Studies of Extracellular Vesicles (MISEV 2023).

### Characterization of EVMs and Tumor-Derived EVs

Nanoparticle Tracking Analysis (NTA) was used to determine the concentration and size of EVMs and EVs using a NanoSight NS300 (Malvern, UK), with five measurements of 60 s each at 25 °C. Western blotting was performed to assess EV markers and peptide binding. Transmission Electron Microscopy (TEM) was used to visualize vesicle structure. EVMs were spotted onto copper grids (3.5–20 × 10^10^ EVs/mL) and stained according to the manufacturer’s instructions using PTK. Images were acquired on a JEOL JEM-1011 transmission electron microscope.

### Western Blot

10^10^ EVs were loaded onto 10% SDS-PAGE (0.375 M Tris, pH 8.8, 0.1% SDS, 10% acrylamide, 0.03% ammonium persulfate (APS), and 0.06% N,N′,N′-tetramethyl ethylenediamine (TEMED)). Membranes were blocked with 5% nonfat milk in 0.1% TBS-Tween for 1 h at room temperature, followed by incubation with primary antibodies overnight at 4 °C and secondary antibodies (SI Table S1) for 1 h at room temperature. Protein bands were visualized with chemiluminescent substrate and visualized in the Bio-Rad Chemi-Doc imaging system.

### Preparation of NANPs

All sequences are listed in the Supporting Information. The Dicer Substrate (DS) RNA sequences^42^ and DNA templates for transcribing RNA strands for functional NANPs (RNA cubes and RNA rings) were purchased from Integrated DNA Technologies (IDT, Coralville, IA, USA). DNA templates were amplified using primers containing the T7 RNA polymerase promoters. Transcription reactions contained 80 mM HEPES-KOH (pH 7.5), 2.5 mM spermidine, 50 mM DTT, 2 mM MgCl_2_, 25 mM rNTPs, 0.2 μM DNA templates, and ∼100 units/μL in-house T7 RNA polymerase, and were incubated at 37 °C for 4–16 h. DNA templates were then degraded with DNase I (Promega) at 37 °C for 30 min. RNA strands were purified using 8 M urea PAGE. RNA cubes and RNA rings were assembled by mixing monomers at equimolar ratios. The mixture was denatured at 95 °C for 2 min. For RNA cubes, samples were transferred to 45 °C and incubated for 2 min, followed by the addition of assembly buffer (2 mM MgCl_2_, 50 mM KCl, 89 mM Tris-borate) and further incubation at 45 °C for 20 min. For RNA rings, samples were snap-cooled on ice for 2 min, assembly buffer was added, and mixtures were incubated at 30 °C for 30 min.

### Physicochemical Characterization of NANPs

To confirm NANP assembly, 8% nondenaturing native-PAGE (37.5:1 Acrylamide/Bis-Acrylamide) was performed in a buffer containing 89 mM Tris-borate (pH 8.2) and 2 mM MgCl_2_. Gels were run at 300 V for 20 min at 4 °C using a Mini-PROTEAN Tetra system (Bio-Rad, Hercules, CA, USA), washed with double-deionized water, and stained with ethidium bromide. NANPs were visualized using a ChemiDoc MP system (Bio-Rad). Atomic force microscopy (AFM) of functional RNA cubes and RNA rings was conducted on freshly cleaved mica surfaces modified with 1-(3-aminopropyl)­silatrane, using a MultiMode AFM Nanoscope IV (Bruker Instruments, Santa Barbara, CA) in tapping mode.^43^

### Loading of EVMs

Synthetic EVMs were loaded with cargo (scramble or anti-GFP DS RNA, or NANPs functionalized with anti-GFP DS RNAs) at an input concentration of 150 nM using four strategies: electroporation, freeze–thaw cycling, passive incubation, and sonication. For electroporation, EVMs were mixed with cargo and subjected to five electrical pulses (∼400 V) in 1 mm-gap cuvettes using an Eppendorf 2510 electroporator, in an acidic buffer (0.02 M citric acid, 0.03 M disodium phosphate, 0.1 mM EDTA, pH 4.4). Samples were then placed on ice for 5 min, briefly vortexed, and incubated at 37 °C for 1 h to allow membrane recovery. For freeze–thaw cycling, EVMs were mixed with cargo and subjected to five cycles of rapid freezing on dry ice (2 min) and thawing at 37 °C (2 min), followed by a 1 h incubation at 37 °C for membrane stabilization. For passive incubation, EVMs and cargo were incubated overnight at 37 °C to allow cargo association. For sonication, EVMs and RNA cargos were subjected to water bath sonication (35 kHz, 30 min; VWR), followed by 1 h incubation at 37 °C. After loading, EVMs were treated with RNase A (400 ng/μL, 37 °C, 30 min) to remove nonencapsulated RNA and purified by ultracentrifugation (100,000*g*, 30–70 min, 4 °C). Pellets were resuspended in PBS for downstream analyses.

### Cell Culture Experiments and 3D Spheroid Formation

Human melanoma cell lines, A375, SKMel-28, and WM-1366; triple-negative human breast cancer cells, MDA-MB-231; and THP-1 Dual human monocytes (a reporter cell line engineered to monitor NF-κB and IRF pathway activation) were cultured at 37 °C in a humidified atmosphere containing 5% CO_2_. SKMel-28 cells were maintained in Minimum Essential Medium (MEM) supplemented with 10% fetal bovine serum (FBS) and 1 mM sodium pyruvate. A375, WM-1366, and MDA-MB-231 cells were cultured in Dulbecco’s Modified Eagle Medium (DMEM) supplemented with 10% FBS; for MDA-MB-231 cells, the medium was further supplemented with 1% penicillin–streptomycin (P/S). THP-1 Dual monocytes (InvivoGen) were maintained according to the manufacturer’s guidelines, alternating between growth medium (RPMI 1640 with 10% FBS, 1% P/S, and normocin 100 μg/mL), selection medium (RPMI 1640 with 10% FBS, 1% P/S, normocin 100 μg/mL, blasticidin 10 μg/mL, and zeocin 100 μg/mL), and test medium (RPMI 1640 with 10% FBS and 1% P/S) for experimental assays. For EV experiments, cells were cultured with EV-depleted FBS, prepared by ultracentrifugation (100,000*g*, 16 h, 4 °C). Monolayers were maintained in standard tissue culture plates. For spheroid formation, 1000–5000 cells/well were seeded onto 96-well plates precoated with a 1% agarose layer (60 μL/well) and centrifuged at 1,150 rcf for 5 min. Fully formed spheroids were observed 72 h later.

### Uptake studies

EV and EVM uptake were evaluated either by lipophilic dye labeling or by monitoring internalization of Alexa Fluor 488-labeled DS RNAs packaged as cargo. For dye-based labeling, EV and EVMs were stained with PKH26 (Sigma-Aldrich) or CellVue Lavender Dye (ThermoFisher) following the manufacturer’s instructions. Briefly, 2 μL of PKH26 or CellVue Lavender dye were diluted in 500 μL of Diluent C and mixed with 500 μL of EV suspension (1:1). The mixture was incubated for 10 min at room temperature, and excess dye was removed by washing with PBS followed by ultracentrifugation at 100,000*g* for 2 h at 4 °C. For DS RNA loading, EVs and EVMs were electroporated with 100 nM Alexa Fluor 488-labeled All Stars Negative Control siRNA (Qiagen), as described above. Stained vesicles were added to 3-day-old spheroids and incubated overnight at 37 °C. The following day, medium containing nonincorporated EVs was removed, spheroids were washed with PBS, imaged under a fluorescence microscope, and dissociated into single-cell suspensions. Uptake was quantified by flow cytometry (Attune NxT, ThermoFisher or Symphony A1, BD Biosciences) by determining the percentage of PKH26^+^, CellVue Lavender^+^ or Alexa Fluor 488^+^ cells. For these experiments, each data point represents the mean percentage of positive cells from eight pooled spheroids.

### Gene Silencing and Toxicity Studies

EVs and EVMs loaded with DS RNAs or NANPs targeting GFP were added to MDA-MB-231-eGFP cells grown as monolayers or spheroids. For monolayers, cells were seeded at 10,000 cells/well in 96-well plates 24 h prior to treatment. For spheroids, 5000 cells/well were seeded in agarose-coated plates and cultured for 3 days before treatment. The RNA concentration of loaded EVs was quantified using Nanodrop2000. Cells were treated once with 15 or 42 nM siRNA, or 2.5 or 7 nM cube or ring NANPs (each containing six siRNAs), maintaining consistent RNA concentrations across loading methods. Positive delivery controls included Lipofectamine 2000 and DOTAP/DOPE (1:10 w/w) complexes incubated with siRNAs or NANPs for 30 min at room temperature before addition to cells. Cells were cultured for 72 h post-treatment, and GFP silencing was assessed by flow cytometry (Attune NxT, Thermo Fisher). GFP inhibition was calculated by normalizing to GFP+ MDA-MB-231-eGFP cells and parental non-GFP MDA cells. For viability assessment, cells from monolayers or dissociated spheroids were washed with PBS and stained with 5 nM Sytox Red in PBS/1% BSA/2 mM EDTA. After 15 min at room temperature, samples were analyzed by flow cytometry (Attune NxT), and the OVERTON analysis tool was used to determine EV uptake and cell toxicity.

## Results

### Characterization and Comparative Analysis of Synthetic EVMs and Tumor-Derived EVs

EVMs were assembled following the protocol that has been established with modifications.^34^ As schematically shown in Figure 1a, multilamellar vesicles (MLVs) were produced from a lipid mixture mimicking the lipid composition found in most natural EVs, which were subsequently processed into small unilamellar vesicles (SUVs) and functionalized with CD63 and CD9 peptides by His-tag-NTA­(Ni) binding. Western blot analysis confirmed the incorporation of CD63 and CD9 in EVMs and compared their levels with ones found in natural melanoma-derived EVs (Figure 1b). Melanoma EVs showed varying expression levels across different cell lines, and importantly, EVMs decorated with CD63 and CD9 peptides (EVM^CD63+/CD9+^) were positive for these markers, indicating successful surface functionalization (Figure 1b). Notably, the lower molecular weight observed for CD63 and CD9 in EVM^CD63+/CD9+^ likely reflects the use of peptides instead of full-length proteins and the absence of post-translational modifications. Furthermore, the absence of the endoplasmic reticulum marker calnexin in melanoma-derived EVs, compared to parental cells, confirmed the purity of the EV preparations and the lack of cellular debris contamination (SI Figure S1a).

**Figure 1:**
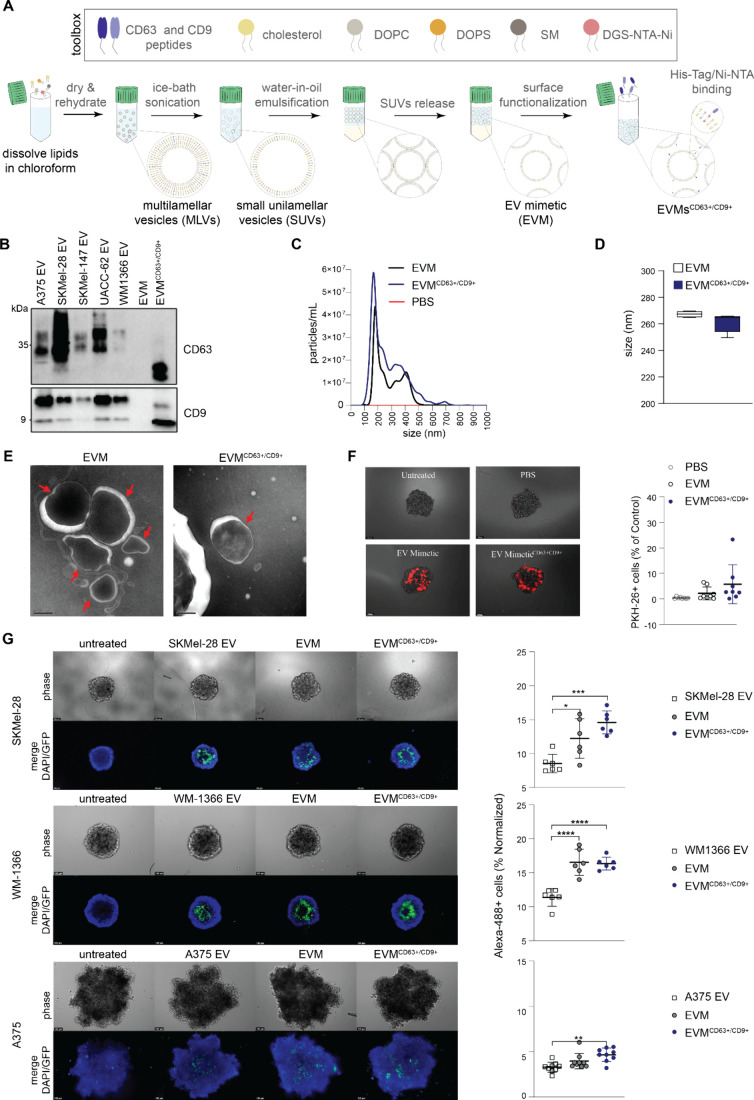
EVMs assembly and characterization. (A) Schematic illustration of the EVM assembly process and surface decoration with His-tagged CD63 and CD9 peptides. (B) Western blot analysis of CD63 and CD9 in natural melanoma-derived EVs and EVMs. (C) EVM profile by NTA (mean, *n* = 5). (D) EVM size by NTA (*n* = 5). (E) TEM images of EVMs (red arrows). Scale bar: 100 nm. (F) Fluorescence microscopy of SKMel-28 spheroids treated with PKH26-labeled EVMs (left) and flow cytometry quantification of PKH26-positive cells after treatment (right). Each value represents a pool of 8 spheroids from 3 independent experiments. PKH26-stained PBS was used as a control. (G) Fluorescence microscopy (left) and flow cytometry quantification (right) of SKMel-28, WM-1366, and A375 spheroids treated with Alexa-488-labeled DS RNAs loaded either in EVMs or in natural EVs. Uptake was normalized to the number of EVs added (1.5–3 × 10^8^ particles/spheroid). Scale bar: 100 μm. Each value represents a pool of 8 spheroids. Statistical analysis was performed using one-way ANOVA followed by a Student’s *t*-test post hoc analysis. Data are presented as mean ± SD and are representative of three independent experiments. ***p < 0.01; ****p < 0.001.

Nanoparticle tracking analysis revealed that EVMs presented a mean size of ∼260–270 nm and a bimodal size distribution (Figure 1c, d), reflecting a heterogeneous vesicle population arising from the assembly process. Transmission electron microscopy further confirmed their membranous, spherical morphology across different sizes (Figure 1e), which was not affected by CD63 and CD9 decoration. More TEM images for EVM^CD63+/CD9+^ are shown in SI Figure S2. In contrast, melanoma-derived EVs were slightly smaller, with average sizes ranging from ∼160 to ∼200 nm, as shown by NTA (SI Figure S1b).

Interestingly, fluorescence microscopy of SKMel-28 spheroids treated with PKH26-labeled vesicles showed distinct uptake patterns, with EVM^CD63+/CD9+^ showing a preferential localization at the periphery compared to lipid-only particles (Figure 1f). Quantitative analysis of cellular uptake was performed by flow cytometry following spheroid dissociation, where each data point represents the average uptake measured from eight pooled spheroids. This analysis revealed that the overall percentage of PKH26-positive cells was similar among the samples (Figure 1f), suggesting that surface decoration influences uptake localization rather than total internalization in SKMel-28 spheroids.

To further assess EVM uptake and intracellular cargo delivery compared to natural melanoma EVs, tumor-derived EVs (SK-Mel-28, WM1366, and A375) and EVMs were loaded with Alexa-488-labeled scramble DS RNA via electroporation and added to three-day-old spheroids. After overnight incubation, fluorescence microscopy showed similar uptake patterns (Figure 1g), while flow cytometry revealed a higher percentage of Alexa-488+ cells for EVMs and EVM^CD63+/CD9+^ compared to natural EVs, likely due to their lack of preexisting cargo facilitating luminal loading (Figure 1g). Uptake values were normalized to vesicle number, as spheroids were treated with equal volumes of EV preparations, though subsequent NTA analysis indicated variable vesicle concentrations among groups.

We next evaluated the effects of melanoma-derived EVs and EVMs on human myeloid cells using THP-1 Dual monocytes as a model. Cells were treated over a three-day period, with vesicles added every 24 h. For clarity, vesicle treatment was initiated at 0 h and repeated at 24 and 48 h. Cell viability was assessed at 24, 48, and 72 h following the initial treatment. Daily vesicle administration was used to model sustained, *in vivo*-like exposure and to ensure comparable treatment conditions between EVs and EVMs. As shown in Figure 2a, treatment with melanoma-derived EVs led to a reduction in viable THP-1 cells over time, as determined by trypan blue exclusion, with EVs from melanoma cell lines derived from primary lesions (A375 and WM-1366) causing the greatest reduction. Lipid-only EVMs also induced a reduction in the number of viable cells at 72 h, while EVM^CD63+/CD9+^ had no significant effect on THP1-Dual cell proliferation.

**Figure 2:**
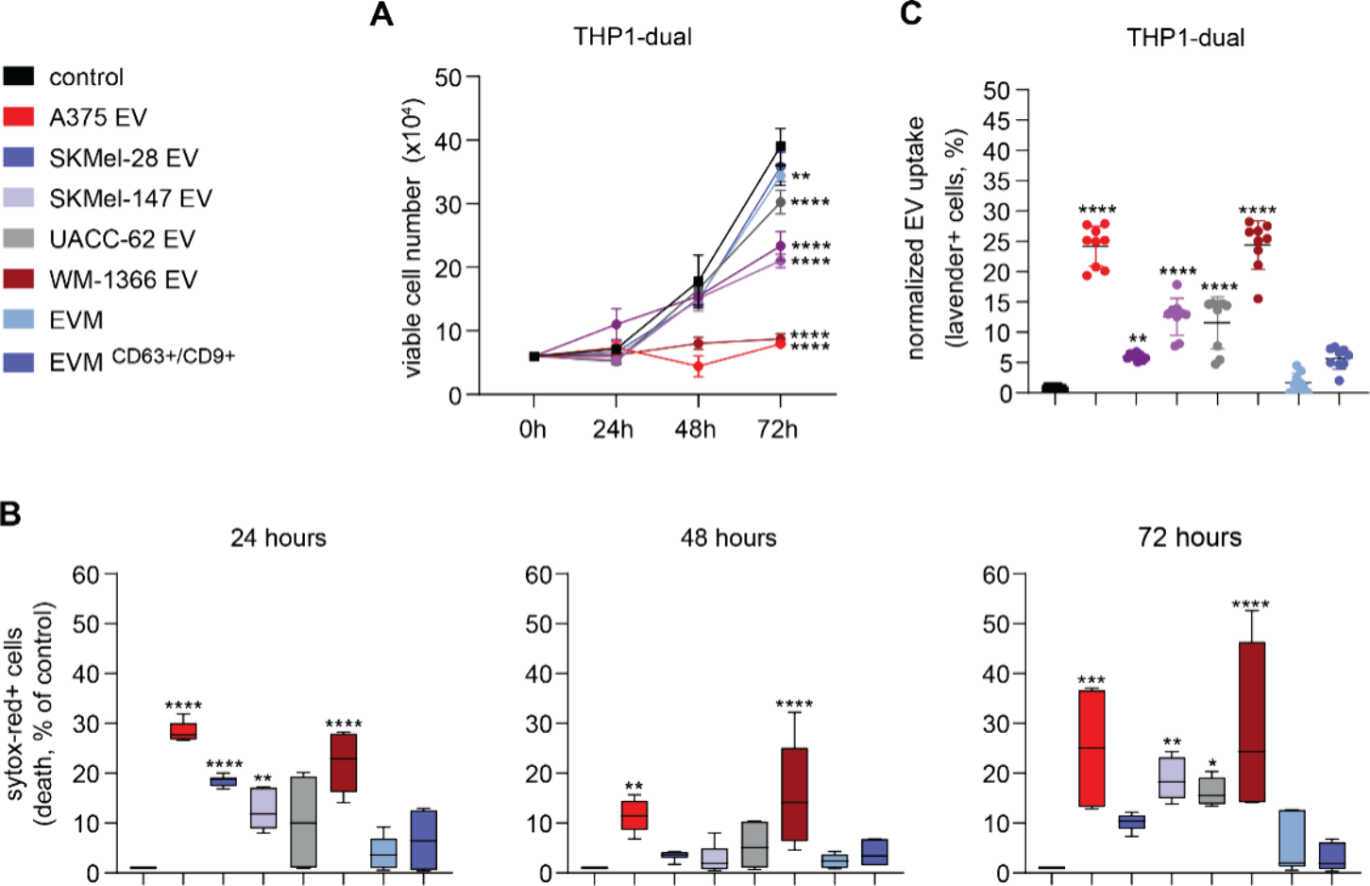
Comparing myeloid cell responses to melanoma EVs and EVMs treatments. (A) Viable cell number of THP-1 dual monocytes over 72 h of treatment with natural melanoma EVs or EVMs. EVs were added 3 times, every 24 h (0.8–1.6 × 10^8^ particles/mL). (B) Cytotoxicity of THP-1 monocytes under the same conditions, assessed by Sytox Red staining and flow cytometry. (C) EV uptake in THP-1 monocytes after overnight incubation with CellVue lavender dye-labeled EVs. *n* = 3 independent experiments. Statistical analysis was carried out using one or two-way ANOVA, with Tukey’s post-test. Data are reported as means ± SD. *p < 0.05; **p < 0.01; ***p < 0.001; ****p < 0.0001. ∗ Indicates statistically significant differences of each sample relative to the control.

Cell viability was further assessed using Sytox Red staining followed by flow cytometry (Figure 2b), which measures loss of plasma membrane integrity as an indicator of cell death. Consistent with the trypan blue exclusion results, natural melanoma EVs induced significant monocyte cell death, which is in linewith their reported immunosuppressive potential.^44–47^ Again, EVs from primary melanoma cell lines were the most cytotoxic across all time points.

EV uptake, assessed by overnight treatment with labeled EVs, showed higher internalization for natural melanoma EVs than for EVMs (Figure 2c), and A375 and WM-1366 EVs presented the highest uptake, which may be associated with higher antiproliferative and cytotoxic effects.

### Influence of RNA Loading Strategy on EVM Incorporation Efficiency

Our next step was to identify the most effective strategy for incorporating different RNAi inducers into EVMs. To address this point, we compared four commonly used loading methods: one passive, incubation at 37 °C, and three active methods: electroporation, freeze–thaw cycling, and water-bath sonication. Three nanostructures targeting GFP via RNAi were evaluated: a Dicer substrate (DS) RNA,^42^ an RNA cube containing six DS RNAs, and an RNA ring containing six DS RNAs. These constructs are designed to release siRNAs against GFP upon intracellular processing by Dicer. EVMs (with or without CD63/CD9 decoration) were loaded with 150 nM of each RNA structure. Electroporation was performed with five pulses of ∼400 V; freeze–thaw consisted of five cycles alternating between dry ice (2 min) and 37 °C (2 min); sonication was carried out for 30 min at 35 kHz; and passive incubation was done overnight at 37 °C. Following active loading, vesicles were incubated for 1 h at 37 °C for membrane recovery, and then all samples were treated with RNase A to degrade unincorporated RNAs, washed with PBS, and ultracentrifuged at 100,000*g* to remove nonvesicular RNA fragments (Figure 3a).

**Figure 3:**
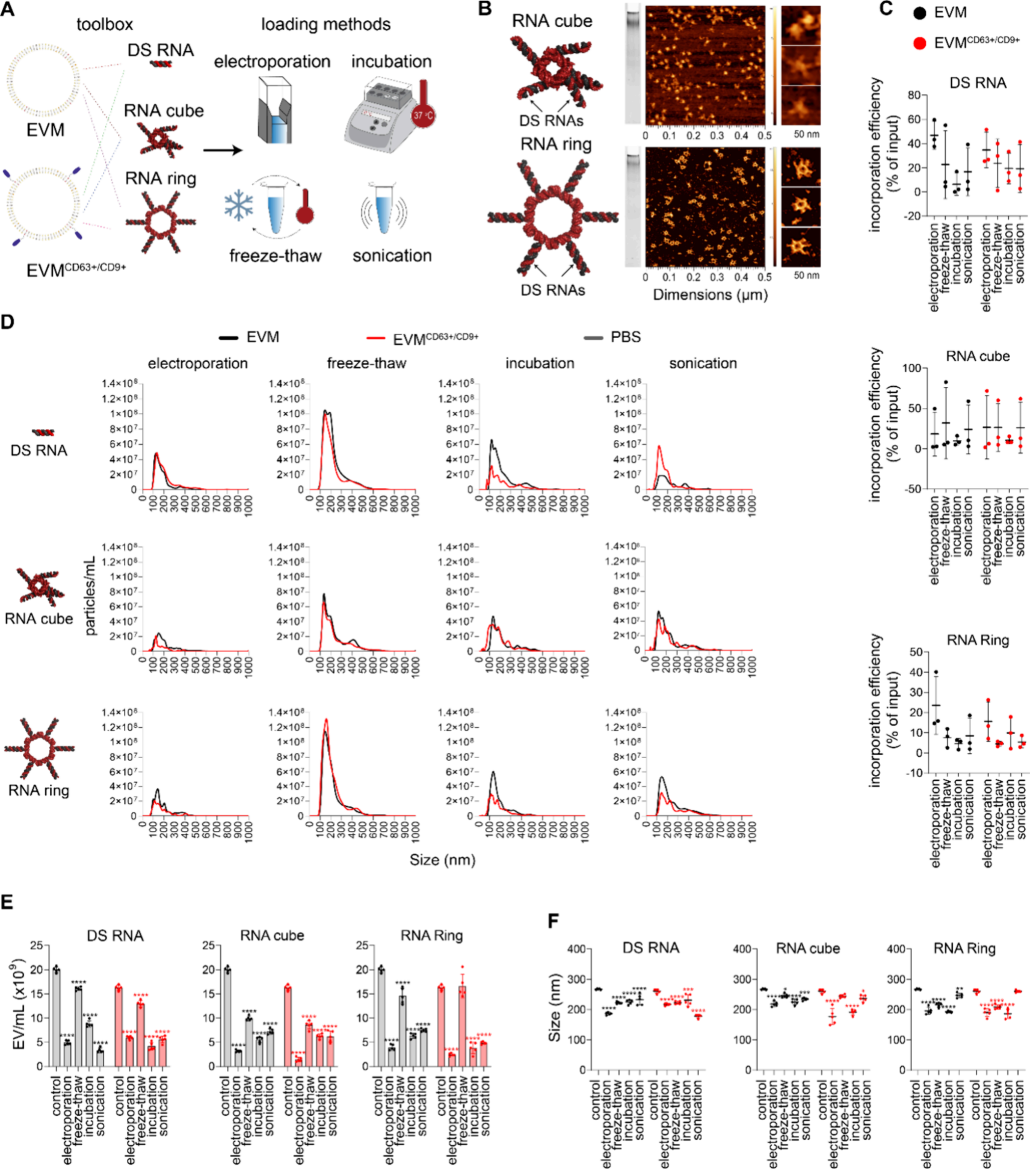
Effect of RNA cargo nanostructure and loading strategy on incorporation efficiency and EVMs profile. (A) Schematic representation of the NANPs’ loading strategies. (B) Representative native-PAGE results and AFM images of RNA cubes and RNA rings functionalized with six anti-GFP DS RNAs. (C) Incorporation efficiency quantified by RNA absorbance before and after loading. *n* = 3 independent experiments. (D) NTA profile of EVMs post loading. Average of 5 measurements. EV-mimetic concentration following RNA loading by NTA (*n* = 5). (E, F) EVMs mean size post loading by NTA (*n* = 5). Statistical analysis was carried out using one-way ANOVA, with Tukey’s post-test. Data are reported as means ± SD **p < 0.01 ***p < 0.001 ****p < 0.0001.

Loading efficiency was determined by RNA absorbance using Nanodrop2000, before and after loading. As shown in Figure 3b, incorporation efficiency varied substantially across independent replicates, consistent with prior observations for small hydrophilic cargo loading into natural EVs.^39^ Importantly, although electroporation caused aggregation of all structures, both DS RNAs and functional NANPs remained stable across the different loading methods, with no evidence of degradation (SI Figure S3).

For DS RNAs, electroporation yielded the highest average incorporation (46.83% ± 9.38 SD for EVM; 34.65% ± 15.8 SD for EVM^CD63+/CD9+^). Freeze–thaw cycling produced intermediate efficiencies (22.62% ± 23.04 SD for EVM; 23.69% ± 16.89 SD for EVM^CD63+/CD9+^). Sonication resulted in lower incorporation (16.68% ± 16.21 SD for EVMs; 19.32% ± 16.99 SD for EVM^CD63+/CD9+^), while passive incubation gave the poorest performance in EVMs (6.35% ± 7.48 SD), and reached levels similar to sonication in EVM^CD63+/CD9+^ (19.45% ± 14.13 SD).

For RNA cubes, the largest three-dimensional structure tested, freeze–thaw gave the highest mean loading efficiency (31.87% ± 35.95 SD for EVMs; 26.34% ± 24.04 SD for EVM^CD63+/CD9+^). Electroporation (18.34% ± 22.18 SD for EVM; 26.6% ± 32.69 SD for EVM^CD63+/CD9+^) and sonication (24.17% ± 24.77 SD for EVM; 26.09% ± 25.77 SD for EVM^CD63+/CD9+^) had moderate efficiencies, all with large deviations. Passive incubation also resulted in the lowest incorporation (9.82% ± 4.6 SD for EVM; 10.52% ± 0.12 SD for EVM^CD63+/CD9+^).

For RNA rings, incorporation was the most consistent among replicates. Electroporation provided the highest efficiency (23.55% ± 11.74 SD for EVM; 15.57% ± 5.82 SD for EVM^CD63+/CD9+^), while freeze–thaw, sonication, and incubation gave comparably lower values ranging from 4 to 10%, with minor differences between undecorated and decorated vesicles.

We next assessed the effect of loading strategies on vesicle concentration and size by NTA. Both decorated and undecorated EVMs exhibited comparable NTA profiles across all loading methods (Figure 3c), which were overall similar to those observed prior to loading (Figure 1c). However, all methods led to a reduction in EV concentration (Figure 3d) and a shift toward smaller average particle size (Figure 3e, f), indicating EVs disruption during incorporation.

### Gene Silencing Efficiency and Toxicity

To evaluate the efficacy of EVM-mediated delivery of RNAi inducers, GFP silencing was assessed in MDA-MB-231-eGFP triple-negative human breast cancer cells cultured as monolayers. EVMs loaded with DS RNA, RNA cubes, or RNA rings using four distinct loading methods were compared. Because RNA cubes and RNA rings each contain six DS RNAs, a 6-fold lower concentration was used relative to free DS RNA to ensure equivalent RNAi inducer content across all treatments.

As illustrated in Figure 4a, MDA-MB-231-eGFP monolayers were plated 1 day prior to treatment in medium containing EV-depleted FBS. EVMs were administered once, 24 h after plating, at two RNA concentrations, with doses adjusted to ensure equivalent siRNA delivery following Dicer-mediated release from DS RNAs and NANPs. GFP silencing and cytotoxicity were assessed by flow cytometry and/or microscopy 72 h post-treatment (Figure 4a and SI Figure S4). Despite equal RNAi inputs, electroporation was the most efficient loading method, particularly at the higher dose (42 nM DS RNA and 7 nM NANPs), achieving GFP silencing comparable to conventional lipid-based carriers (Lipofectamine 2000 and DOTAP/DOPE; Figure 4b). In contrast, the other loading strategies resulted in only minimal silencing activity.

**Figure 4:**
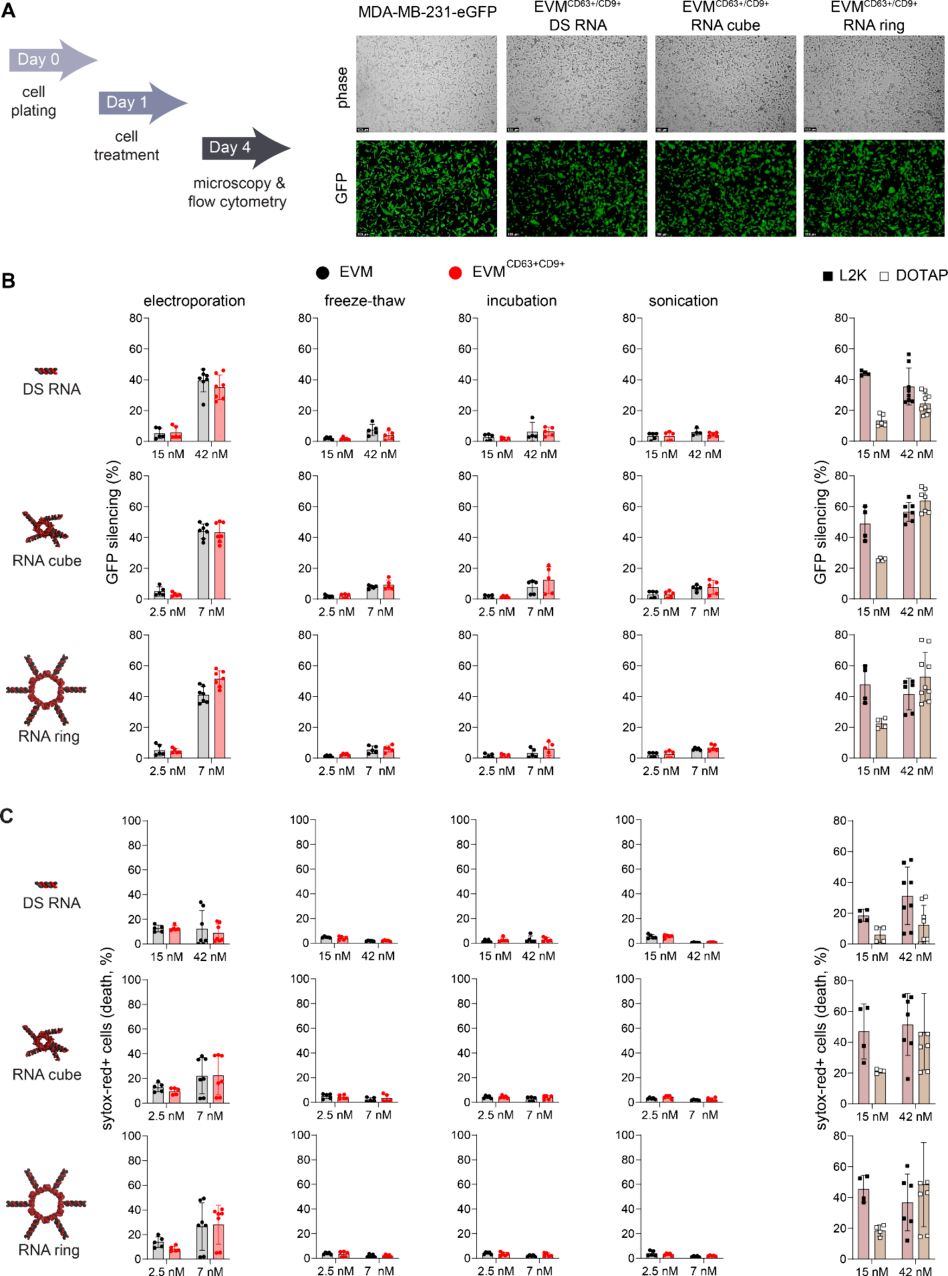
Effect of RNA cargo nanostructure and loading strategy on GFP silencing efficiency and cytotoxicity of EVMs in monolayer culture. (A) Experimental design (left). Cells were plated in EV-depleted medium and treated 24 h later with EVMs or lipid-based controls (L2K, DOTAP/DOPE) at RNA doses normalized to equal concentration across nanostructures and loading methods. Representative microscopy of untreated and treated samples on day 4, 72 h after treatment (right). Scale bar: 100 μm. (B) GFP silencing quantified by flow cytometry 72 h after treatment. (C) Cytotoxicity analysis by Sytox Red staining and flow cytometry 72 h after treatment. *n* = 3 independent experiments. Data are reported as mean ± SD.

Among the different RNA nanostructures, silencing efficiency was overall similar, with RNA rings displaying a modest increase in GFP silencing, particularly in EVM^CD63+/CD9+^. In terms of cytotoxicity, assessed by Sytox-Red staining, EVM exhibited lower toxicity compared to L2K and DOTAP/DOPE (Figure 4c). Notably, these cationic carriers exhibited intrinsic cytotoxicity, which was further enhanced when combined with NANPs or DS RNA, whereas EVMs alone showed no significant induction of cell death (Figure 4c, SI Figure S5).

Because translation of *in vitro* efficacy to *in vivo* settings is constrained by tissue complexity and limited nanoparticle penetration in three-dimensional environments, we next evaluated GFP silencing in MDA-MB-231-eGFP spheroids. Spheroids were allowed to form for 3 days prior to treatment with EVMs at the same concentrations used in monolayer experiments. GFP silencing and cytotoxicity were assessed on day six, corresponding to 72 h post-treatment. (Figure 5a, SI Figure S6). As expected, silencing efficiency was generally lower in spheroids compared to monolayers. However, electroporation-loaded EVMs outperformed lipid carriers, achieving higher GFP silencing across all RNA structures (Figure 5b). Cytotoxicity analysis in spheroids, performed after dissociation and Sytox-Red staining, revealed that mimetics induced a toxicity profile similar to lipid carriers, but with higher silencing efficacy (Figure 5c). Interestingly, similar to monolayer-cultured cells, DS RNAs showed lower toxicity compared to NANPs, which may be attributed to IRF activation, and could be advantageous in a therapeutic context.

**Figure 5:**
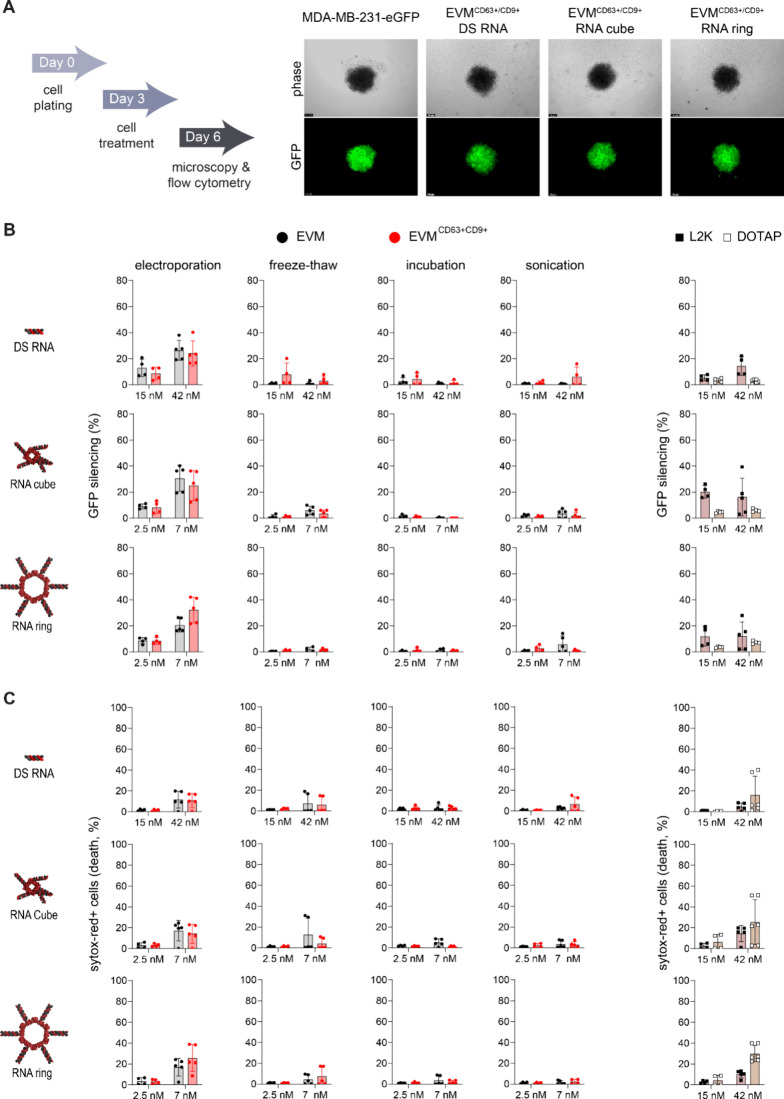
Effect of RNA cargo nanostructure and loading strategy on GFP silencing efficiency and cytotoxicity of EVMs in spheroids. (A) Experimental design (left). Spheroids were plated in agarose-coated 96-well plates, cultured in EV-depleted medium, and treated 72 h later with EVMs or lipid-based controls (L2K, DOTAP/DOPE) at RNA doses normalized to equal concentration across nanostructures and loading methods. Representative microscopy of untreated and treated samples on day 6, 72 h after treatment (right). Scale bar: 100 μm. (B) GFP silencing quantified by flow cytometry 72 h after treatment. (C) Cytotoxicity analysis by Sytox Red staining and flow cytometry 72 h after treatment. *n* = 3 independent experiments. Data are reported as mean ± SD.

## Discussion

EVs are now appreciated as powerful mediators of intercellular communication.^48^ Their intrinsic ability to deliver functional biomolecules to specific target cells, including proteins, lipids, and nucleic acids, has positioned them as promising candidates for next-generation drug delivery systems.^49^ Particularly, EVs are increasingly explored as therapeutic carriers for RNA-based drugs, offering advantages over other nanocarriers, such as biocompatibility, low immunogenicity, and enhanced cargo transfer efficiency.^25,28,31,50^

Despite their potential, clinical translation of EVs remains limited by challenges in large-scale production, efficient cargo loading, and functional engineering.^33^ Although preclinical studies have demonstrated promising results for RNAi-based therapeutics using EV carriers both *in vitro* and *in vivo*, achieving consistent and efficient cargo encapsulation remains a major obstacle.^39,51^

Active loading methods, such as electroporation, freeze–thaw cycling, and sonication, are commonly used, resulting in transient membrane permeability and enabling the loading of exogenous cargo.^52,53^ Among them, electroporation is the most used, causing EV permeabilization by the application of electric fields and leading to loading efficiencies ranging from 0.05% to 60%.^54^ Sonication is the second most used strategy and induces mechanical disruption via cavitation, resulting in encapsulation efficiencies from 8% to 30%.^54^ Freeze–thaw cycling, although less frequently used, has shown good loading capacity by inducing membrane permeabilization through the formation of ice crystals, with efficiencies ranging from 3 to 55%.^39,51^ Passive incubation, on the other hand, is generally effective for hydrophobic cargo^55^ but less efficient for hydrophilic molecules, usually resulting in loading efficiencies less than 10%.^39,56^

The fact that most loading methods show overall low and highly variable efficiency can be related to multiple parameters, including EV composition, cargo biochemical properties, and loading parameters such as the number of loading cycles and duration.^54^ Moreover, the intrinsic complexity of natural EVs, including their endogenous cargo and surface content may further influence therapeutic cargo incorporation. This, together with their low production yield and challenging functionalization, has been limiting their therapeutic use as carriers.

In this context, the development of synthetic EVMs offers the advantage of lacking cell-specific tropism and cargo, and allowing straightforward functionalization, as well as scalable production. Bottom-up assembly has been shown of the fully synthetic vesicles resembling EV lipid composition and functionalization with tetraspanins CD63 and CD9,^34^ shown to play important roles in EVM internalization and cargo delivery.^35,36^ These synthetic vesicles successfully delivered functional siRNAs for wound-healing therapy, supporting the feasibility of EV-inspired synthetic delivery systems.

In oncology, RNAi-based therapies can enhance treatment efficacy by targeting disease-associated pathways and overcoming intrinsic and acquired resistance mechanisms. However, tumor resistance is often multifactorial, frequently requiring combinatorial strategies. In this scenario, the use of NANPs offers important advantages as they can simultaneously deliver multiple identical or different DS RNAs within a single particle, enabling Disser-assisted siRNA release and multitarget gene silencing, while also acting as immune stimulatory agents via RIG-I activation and IRF signaling induction.^19–21^ This dual gene-silencing and immune-activation approach has recently been explored,^57^ where red blood cell-derived EVs carrying both a KRAS-targeted antisense oligonucleotide and a RIG-I agonist were used to treat colorectal and nonsmall cell lung cancer (NSCLC), resulting in enhanced antitumor immunity and prolonged survival in preclinical models.

Here, we developed EVM platform inspired by established protocols,^34^ incorporating an EV-like lipid composition and the canonical EV markers CD63 and CD9, which were coupled to EVM membranes via Ni^2+^-NTA/His-tag binding. To investigate their potential for RNAi-based drug delivery to tumors, melanoma spheroids were treated with EVMs carrying Alexa-488-labeled RNAs. EVM-treated spheroids exhibited a higher percentage of fluorescent cells compared with spheroids treated with natural melanoma-derived EVs loaded with the same RNA concentration. This reflects more efficient cargo loading, as synthetic mimetics lack competing endogenous cargo that may otherwise limit luminal loading. Moreover, the modular binding strategy used to couple CD63 and CD9 enables straightforward functionalization, facilitating cell-specific delivery and modulation of EVM tropism.^37,58^

To evaluate safety and immunological interactions, THP-1 monocytes were treated with melanoma-derived EVs and EVMs for 3 days. Melanoma EVs presented an antiproliferative and cytotoxic effect on immune cells, importantly, not seen for EVM^CD63+/CD9+,^ highlighting their potential clinical use in oncology. Interestingly, uptake analysis revealed that EVMs were more efficient in targeting melanoma cells, whereas natural EVs showed higher uptake by monocytes. This cell-type dependent behavior suggests that EVMs are suitable for tumor-targeted delivery, possibly presenting minimal off-target interactions with immune cells.

Finally, we evaluated functional RNA delivery in MDA-MB-231-eGFP cells. EVMs were loaded with GFP-targeting cargos, and multiple loading strategies were compared to identify optimal conditions for different functional architectures. Consistent with previous reports for natural EVs,^39^ we observed substantial inter-replicate variability. Among the tested nanostructures, RNA rings exhibited the most consistent loading, likely due to planar geometry of these NANPs, which may favor membrane interaction and vesicle encapsulation. Notably, despite equivalent RNA input, electroporation produced the highest level of functional gene silencing, comparable to Lipofectamine 2000 and DOTAP/DOPE, but with reduced toxicity in monolayer cultures. In 3D spheroid models, electroporation-loaded EVMs outperformed conventional lipid carriers while maintaining similar toxicity profiles, underscoring their translational potential for targeting resistant tumor cells. Although the other loading methods achieved measurable RNA association, they produced minimal silencing, raising questions about whether RNA was incorporated luminally in a functional state or remained membrane-bound and/or a nonreleasable or functional form. Due to individual vesicles carrying only small amounts of RNA, we were unable to confirm RNA encapsulation via gel electrophoresis, representing a technical limitation of this study. However, free DS RNAs and functional NANPs subjected to the different loading methods showed no evidence of degradation, indicating that their structural integrity was preserved during loading.

## Conclusion

Overall, this study demonstrates the strong potential of synthetic EVMs as programmable nucleic acid delivery platforms in advanced cancer models. In direct comparison to tumor-derived EVs, EVMs effectively encapsulated and functionally delivered diverse RNAi-inducing architectures, achieving robust gene silencing in both tumor monolayer and spheroid cultures, with performance comparable to or exceeding that of conventional lipid carriers. EVMs exhibited a higher delivery capacity to melanoma spheroids than tumor-derived EVs and showed no effect on monocyte proliferation or viability. Our results highlight the modularity and tunability of EVMs, suggesting they can be engineered to optimize tissue targeting, cargo loading, and functional outcomes. Together, these findings support the potential of EVMs as a safe and versatile delivery platform for next-generation RNAi therapeutics.

## Supplementary Material



## Notes

Nucleic acid sequences used in this study; additional physicochemical characterization of EVMs (Figures S1–S2); stability analysis of different loading strategies (Figure S3); cell viability assessments; 2D and 3D GFP silencing studies (Figures S4–S6). Antibodies and dilutions used for Western blot (Table S1. (PDF)
