# Supplementary material for: Engineering Optimized EV-Mimetic Carriers for Efficient Tumor-Targeted Delivery of Functional RNA Nanoparticles
Source: ACS Nano Med. 2026 Feb 20;1(5):1310–22. doi: 10.1021/acsnanomed.6c00004 (PMC12965198; doi:10.1021/acsnanomed.6c00004)
Supplement: Supplementary file 1 [file nm6c00004_si_001.pdf]

Supporting Information

Engineering Optimized EV-Mimetic Carriers for Efficient Tumor-Targeted Delivery of Functional RNA Nanoparticles

Nathalia Leal Dovaizem[1,2,3], Laura P. Rebolledo[3], Anh Ha[3], Roger Chammas[1,2], Luciana Nogueira de Sousa Andrade[1,2# *]and Kirill A. Afonin[3#*]

[1] Center for Translational Research in Oncology (LIM24), Instituto do Câncer do Estado de São Paulo, Hospital das Clínicas da Faculdade de Medicina da Universidade de São Paulo.
[2] Comprehensive Center for Precision Oncology, Universidade de São Paulo, São Paulo 01246-000, Brazil.
[3] Department of Chemistry, University of North Carolina at Charlotte, Charlotte, North Carolina 28223, United States.

# Co-senior authors
*Correspondence to Kirill A. Afonin at kafonin@charlotte.edu and Luciana Nogueira de Sousa Andrade at luciana.nsandrade@hc.fm.usp.br

Sequences used in this project

Dicer Substrate (DS) RNA against GFP[1]
sense
5’-pACCCUGAAGUUCAUCUGCACCACCG
antisense
5’-CGGUGGUGCAGAUGAACUUCAGGGUCA

RNA Cube functionalized with six copies of DS RNAs against GFP.
A:GGCAACUUUGAUCCCUCGGUUUAGCGCCGGCCUUUUCUCCCACACUUUCACGUUCGG
UGGUGCAGAUGAACUUCAGGGUCA
B:GGGAAAUUUCGUGGUAGGUUUUGUUGCCCGUGUUUCUACGAUUACUUUGGUCUUCG
GUGGUGCAGAUGAACUUCAGGGUCA
C:GGACAUUUUCGAGACAGCAUUUUUUCCCGACCUUUGCGGAUUGUAUUUUAGGUUCGG
UGGUGCAGAUGAACUUCAGGGUCA
D:GGCGCUUUUGACCUUCUGCUUUAUGUCCCCUAUUUCUUAAUGACUUUUGGCCUUCGG
UGGUGCAGAUGAACUUCAGGGUCA
E:GGGAGAUUUAGUCAUUAAGUUUUACAAUCCGCUUUGUAAUCGUAGUUUGUGUUUCGG
UGGUGCAGAUGAACUUCAGGGUCA
F:GGGAUCUUUACCUACCACGUUUUGCUGUCUCGUUUGCAGAAGGUCUUUCCGAUUCGG
UGGUGCAGAUGAACUUCAGGGUCA
sense
5’-pACCCUGAAGUUCAUCUGCACCACCG

RNA Ring functionalized with six copies of DS RNAs against GFP.
A:GGGAACCGUCCACUGGUUCCCGCUACGAGAGCCUGCCUCGUAGCUUCGGUGGUGCA
GAUGAACUUCAGGGUCA
B:GGGAACCGCAGGCUGGUUCCCGCUACGAGAGAACGCCUCGUAGCUUCGGUGGUGCA
GAUGAACUUCAGGGUCA
C:GGGAACCGCGUUCUGGUUCCCGCUACGAGACGUCUCCUCGUAGCUUCGGUGGUGCA
GAUGAACUUCAGGGUCA
D:GGGAACCGAGACGUGGUUCCCGCUACGAGUCGUGGUCUCGUAGCUUCGGUGGUGCA
GAUGAACUUCAGGGUCA
E:GGGAACCACCACGAGGUUCCCGCUACGAGAACCAUCCUCGUAGCUUCGGUGGUGCAG
AUGAACUUCAGGGUCA
F:GGGAACCGAUGGUUGGUUCCCGCUACGAGAGUGGACCUCGUAGCUUCGGUGGUGCA
GAUGAACUUCAGGGUCA
sense
5’-pACCCUGAAGUUCAUCUGCACCACCG

Supporting Figures

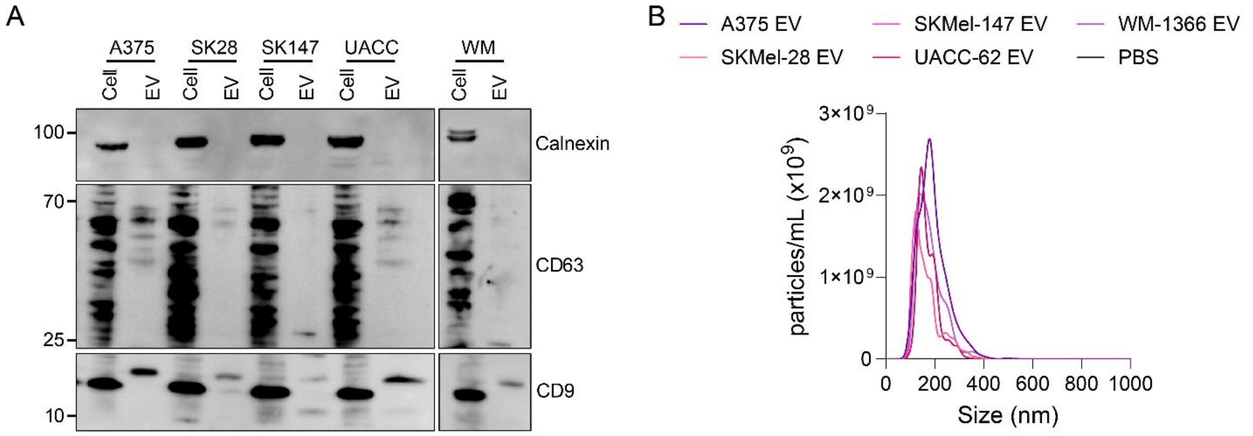

Figure S1. Characterization of melanoma-derived EVs. **A)** Western blot showing the absence of cellular debris contamination in EV pellets, confirmed by the lack of the endoplasmic reticulum marker calnexin**. B)** NTA profile of melanoma-derived EVs obtained using NanoSight NS3000 (mean of five measurements).

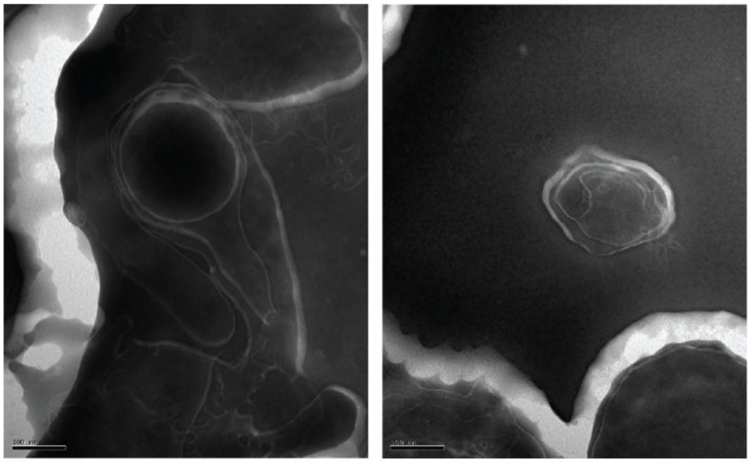

Figure S2. Additional TEM images of EVMs with His-tagged CD63 and CD9 peptides.

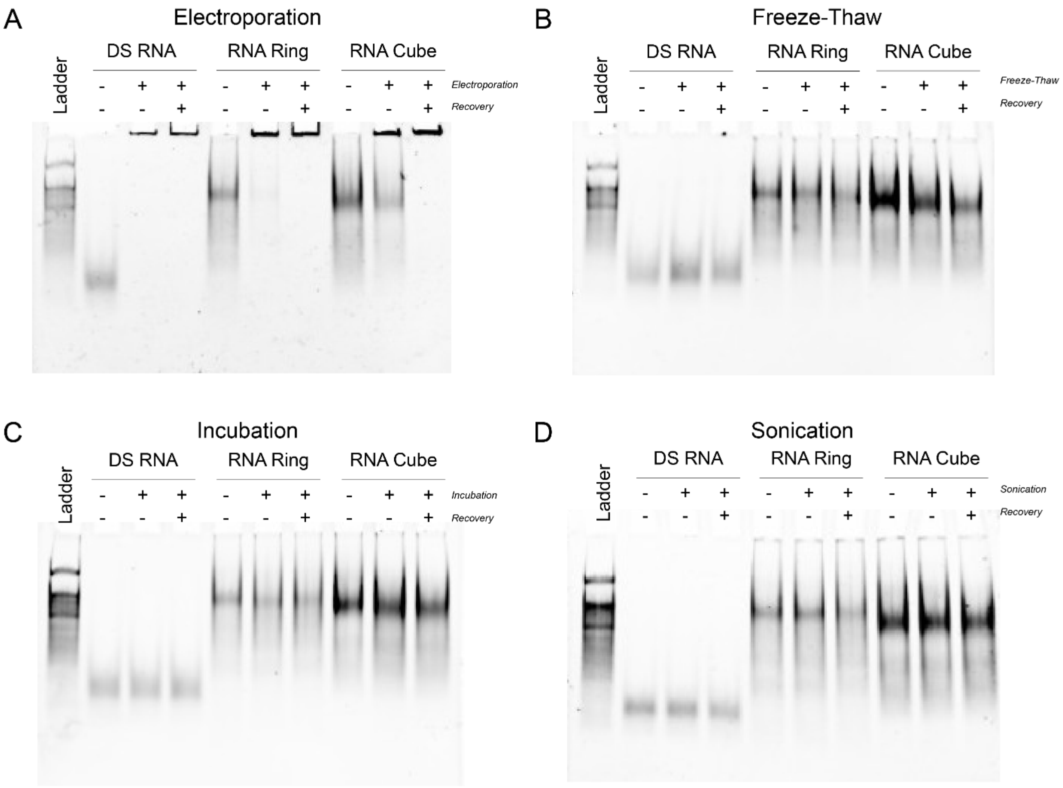

Figure S3. A stability analysis of DS RNA and NANPs in the absence of EVs following the same protocols used for various loading strategies, assessed by 5% native-PAGE. All recoveries were performed at 37 °C for 1 h. Panels **A**)–**D**) correspond to electroporation, freeze–thaw, incubation, and sonication, respectively.

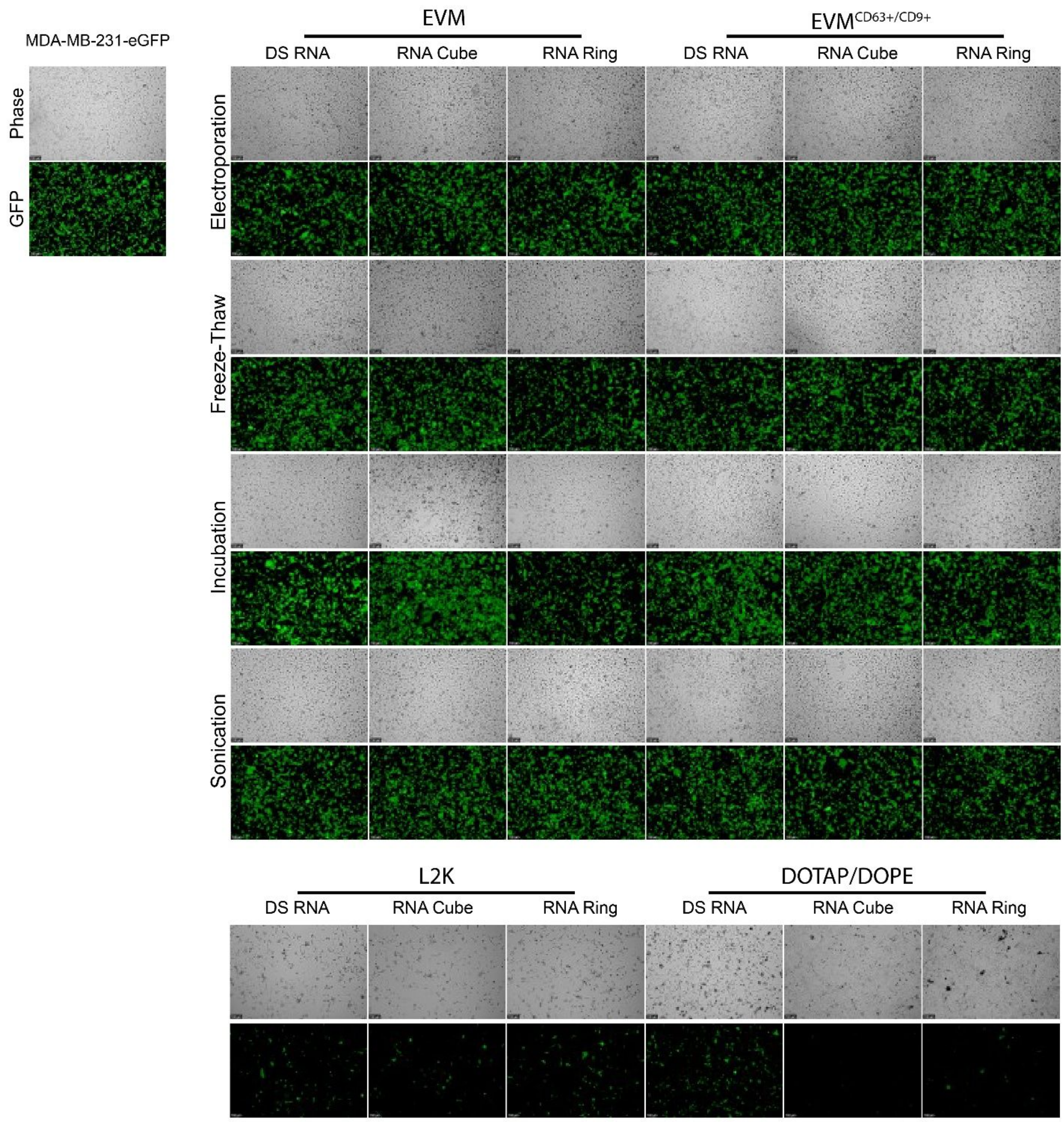

Figure S4. Fluorescence microscopy of GFP⁺ cells in monolayers 72 h post-treatment. MDA-MB-231-eGFP cells were treated with GFP-targeting DS RNA, RNA Cubes, or RNA Rings delivered *via* EVMs, L2K, or DOTAP/DOPE (42 nM duplex; 7 nM Cube or Ring). Fluorescence images were acquired 72 h after treatment.

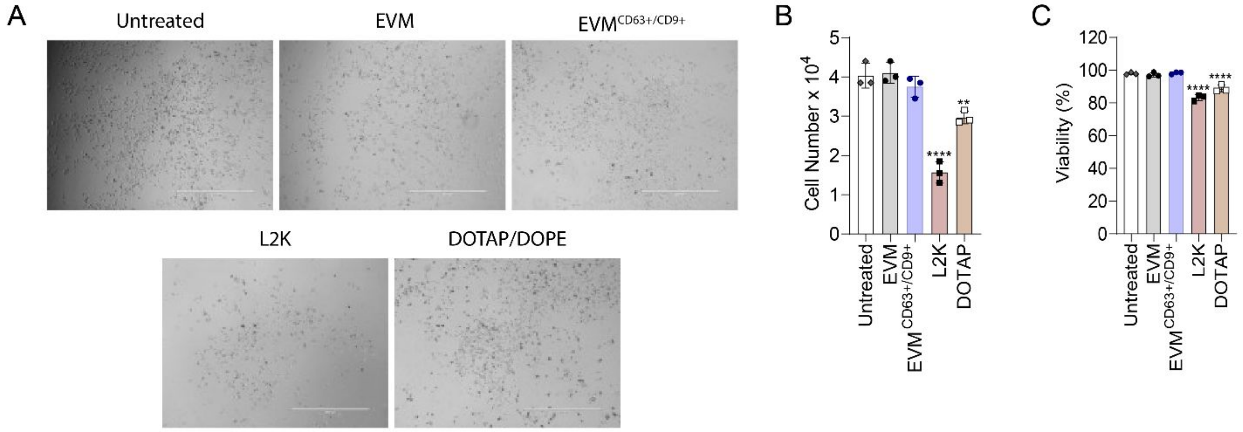

Figure S5. Effect of carrier-only treatment on MDA-MB-231 cells viability. **A**) Microscopy of MDA-MB-231 cells 72 h after treatment with carriers only (Scale Bar: 1000 μm). **B**) Total cell number 72 h after treatment, by trypan-blue count. **C)** Cell viability obtained by trypan blue count. Statistical analysis was done using one-way ANOVA using Bonferroni post-test. Data are reported as mean ± SD (**p<0.01 **** p < 0.0001. *). Statistical significance is indicated for each sample relative to the control.

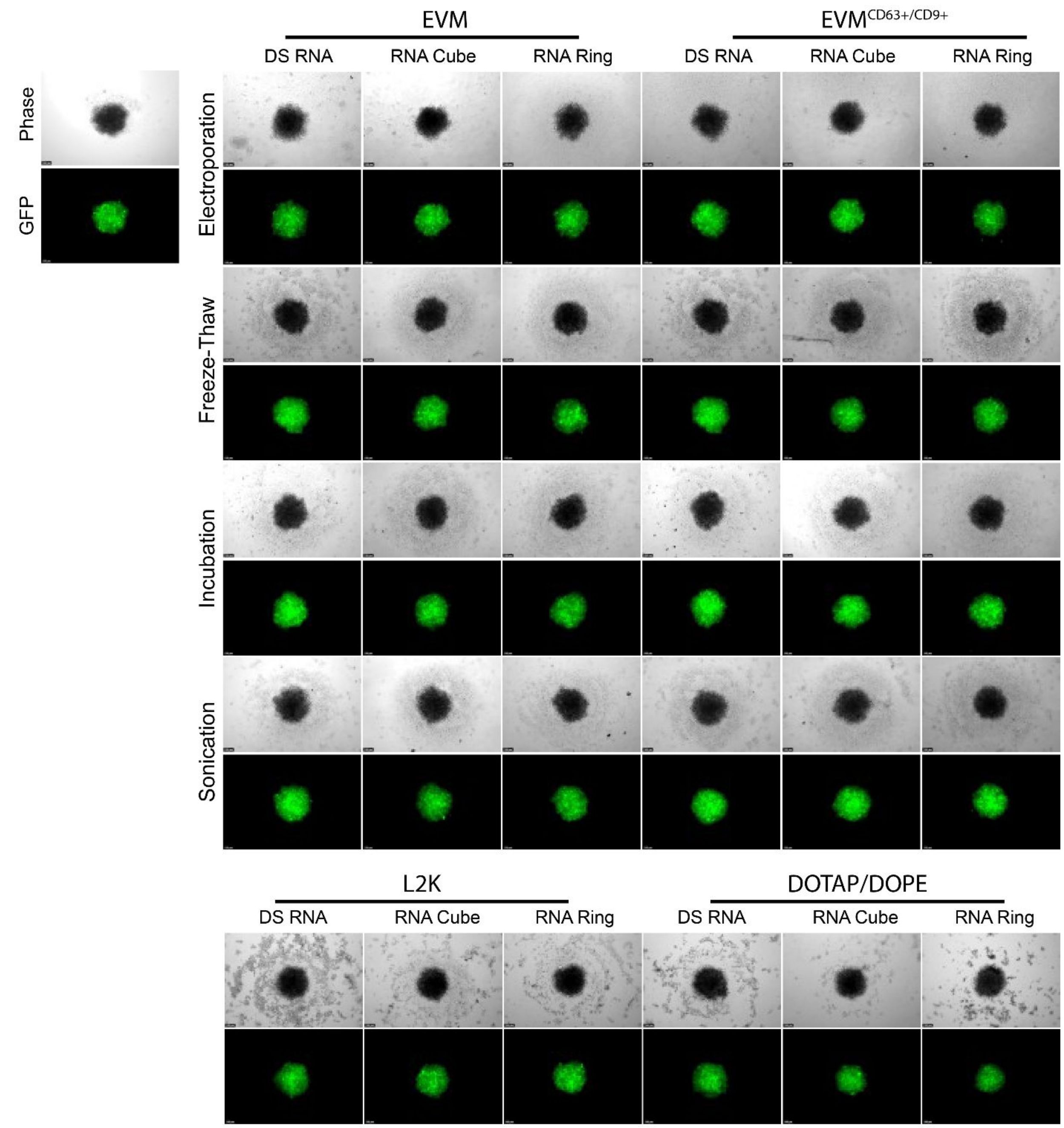

Figure S6. Fluorescence microscopy of MDA-MB-231-eGFP cells in spheroids 72 h post-treatment. Three-day-old MDA-MB-231-eGFP spheroids were treated with GFP-targeting DS RNA, RNA Cubes, or RNA Rings delivered via EVMs, L2K, or DOTAP/DOPE (42 nM duplex; 7 nM Cube or Ring). Fluorescence images were acquired 72 h after treatment.

Supporting Tables

Table S1. Antibodies and dilutions used for Western blot

Antibody	Dilution (titer)
Anti-Calnexin (Abcam - ab58504)	1:500
Anti-CD63 (Thermo Fisher - PA592370)	1:700
Anti-CD9 (Santa Cruz - SC13118)	1:100
Anti-rabbit IgG HRP (Abcam - ab205718)	1:5000
Anti-mouse IgG HRP (Cell Signaling - 707656s)	1:2000

Supporting Reference:

(1) Rose, S. D.; Kim, D. H.; Amarzguioui, M.; Heidel, J. D.; Collingwood, M. A.; Davis, M. E.; Rossi, J. J.; Behlke, M. A. Functional polarity is introduced by Dicer processing of short substrate RNAs. *Nucleic acids research* **2005**, *33* (13), 4140-4156.
